# 

**Published:** 1844-07

**Authors:** 


### CONTENTS

OF NO. VII.

---

### ORIGINAL COMMUNICATIONS.

### ESSAYS AND CASES.

Art. I.—The Replier replied to, and the Reviewer reviewed; a letter to Lunsford P. Yandell, M.D., containing Strictures and Thoughts on the errors and false doctrines of Chemico-Physiology. By Carles Caldwell, M.D. - - - - - - - - - - 1

Art. II.—On the use of Stramonium in Laryngismus Stridulus. By Dr. J. P. Epperson, of Pulaski, Tennessee. - - - - - - - - - - - 25

Art. III.—Extensive Disease of the Gums and Alveoli, from a decayed tooth. By Samuel Griffith, Surgeon Dentist, of Louisville, Ky. - - - - - 28

### REVIEWS.

Art. IV.—Outlines of Pathology and Practice of Medicine. By William Pulteney Alison, M. D., F. R. S., Fellow and late President of the Royal College of Physicians, Edinburgh; Professor of the Practice of Medicine in the University of Edinburgh, &c., &c. - 30

Art. V.—The Cyclopædia of Practical Medicine. Edited by John Forbes, M.D., F.R.S., Alex. Tweedie, M.D., F.R.S., and John Conolly, M.D. Revised, with additions, by Robley Dunglison, M.D. - - 45

Art. VI.—A Treatise on Operative Surgery, comprising a description of the various processes of the art, including all the new operations; with 80 plates, containing 486 separate illustrations. By Jos. Pancoast, M.D., Professor of General, Descriptive, and Surgical Anatomy in the Jefferson Medical College, Philadelphia, &c., &c. - - - - - - - 51
Art. VII.—A Practical Treatise on the Diseases of the Testis, and of the Spermatic Cord and Scrotum, with illustrations. By T. B. Curling, Lecturer on Surgery and Assistant Surgeon to the London Hospital; Surgeon to the Jews' Hospital, &c. Edited by P. B. Goddard, M.D., M.A.P.S., M.A.N.S., Demonstrator of Anatomy in the University of Pennsylvania, &c. - - - - - - - - 53

SELECTIONS FROM AMERICAN AND FOREIGN JOURNALS.

Cases of Longevity no test of the average duration of man's life. - - - - - - - - - - 64
Longevity greatest among the poor. - - - - - 65
Extraordinary Case of Vicarious Menstruation. - - - 65
On the use of Mustard in the Convulsions of Children. - 66
Nitrate of Silver in Diseases of the Eye. - - - - 67
Case of Dysphonia. - - - - - - - - 67
Belladonna in Dysmenorrhœa and Neuralgia. - - - 68
Spermatorrhœa. - - - - - - - - - 70
Some account of an Hysterical Affection of the Vocal Apparatus, with several Cases. - - - - - 75
Epidemic Ague, or Fainting Fever of Persia. - - - 76
On the Temperature of Children. - - - - - 76
Operation for Hare-Lip. - - - - - - - 77
Restored Vision. - - - - - - - - - 77
Nervous Headache. - - - - - - - - 78
Hydriodate of Potash. - - - - - - - 78
Erysipelas. - - - - - - - - - - 78
Arsenic in Diseases of the Skin. - - - - - 79
Nature and Treatment of Hæmoptysis. - - - - 81
Doctors. - - - - - - - - - - 82
Sciatica—Iodide of Potassium. - - - - - - 84

ORIGINAL INTELLIGENCE.

Professor Caldwell and Animal Chemistry. - - - - 85
Dunglison's Practice of Medicine. - - - - - 91
Western Enterprise. - - - - - - - - 91
Transylvania Medical School. - - - - - - 92

